# Large-Scale Item-Level Analysis of the Figural Matrices Test in the Norwegian Armed Forces: Examining Measurement Precision and Sex Bias

**DOI:** 10.3390/jintelligence12090082

**Published:** 2024-08-29

**Authors:** Fredrik Helland-Riise, Tore Nøttestad Norrøne, Björn Andersson

**Affiliations:** 1 Centre for Educational Measurement (CEMO), University of Oslo, 0318 Oslo, Norway; bjorn.andersson@cemo.uio.no; 2 The Norwegian Armed Forces, 0593 Oslo, Norway; tore.norrone@uit.no; 3 Faculty of Health Science, UiT The Arctic University of Norway, 9037 Tromsø, Norway; 4 Centre for Research on Equality in Education (CREATE), University of Oslo, 0318 Oslo, Norway

**Keywords:** fluid intelligence, figural matrices, measurement precision, sex bias, measurement invariance, item response theory

## Abstract

Figural matrices tests are common in intelligence research and have been used to draw conclusions regarding secular changes in intelligence. However, their measurement properties have seldom been evaluated with large samples that include both sexes. Using data from the Norwegian Armed Forces, we study the measurement properties of a test used for selection in military recruitment. Item-level data were available from 113,671 Norwegian adolescents (32% female) tested between the years 2011 and 2017. Utilizing item response theory (IRT), we characterize the measurement properties of the test in terms of difficulty, discrimination, precision, and measurement invariance between males and females. We estimate sex differences in the mean and variance of the latent variable and evaluate the impact of violations to measurement invariance on the estimated distribution parameters. The results show that unidimensional IRT models fit well in all groups and years. There is little difference in precision and test difficulty between males and females, with precision that is generally poor on the upper part of the scale. In the sample, male latent proficiency is estimated to be slightly higher on average, with higher variance. Adjusting for measurement invariance generally reduces the sex differences but does not eliminate them. We conclude that previous studies using the Norwegian GMA data must be interpreted with more caution but that the test should measure males and females equally fairly.

## 1. Introduction

The mass testing of individuals using standardized tests is a common method to map or screen ability for pedagogical, diagnostic, or selection purposes. One such test battery is the General Mental Ability (GMA) test of the Norwegian Armed Forces (NAFs), which is well known in the field of intelligence, and it has been used to make scientific statements about the Norwegian population (Sundet et al. 2004), as well as intelligence in general (Flynn 1987; Flynn and Shayer 2018).

The Norwegian GMA tests were, in their time, developed for males, but they have later been utilized to measure both sexes, though with a lack of validation. This study sought to address this by investigating the measurement properties of the figural matrices test—the arguably most important subtest of the battery. A detailed analysis of the measurement properties of the items in the test using item response theory (IRT) had not been done before. This is a crucial analysis for understanding both how the instrument obtains measurements in general, as well as how it works for women. Our research suggests that this has consequences both for practical use in selection and classification, as well as for the multitude of research that has been conducted using the Norwegian GMA measures.

### 1.1. General Mental Ability Testing

GMA is a psychological construct inferred via a battery of cognitive tests that are intended to measure a person’s latent mental abilities relative to others in the population (Jensen 1998). The score from a GMA battery is recognized as a generally valid predictor of job performance and training proficiency across multiple occupations and cultures (Sackett et al. 2022). The tests are especially attractive for personnel selection because of an easy administrative process and a low application cost, making it ideal for screening large groups of people.

The NAFs have used GMA testing to select personnel since the first test was developed in 1954 (Hansen 2006), and the battery consists of a numeracy test (U4), a figural matrices test (U5), and a word similarities test (U6). We will refer to the U5 as the NAF figural matrices for the remainder of this article. The sum scores from the tests are aggregated and transformed to a normed, standard 1–9 (stanine) scale. The U4 has undergone some changes over the years, but the U5 and U6 tests have been used relatively unchanged with occasional re-norming. The GMA testing of the NAFs is based on the idea that just using a few different measures is sufficient to extract a general mental ability score, which is a practice that is supported by, e.g., Gustafsson (1984); Jensen and Weng (1994).

Figural matrices tests are a central part of GMA testing due to their high score correlation with scores of a general ability factor (Snow 1981), correlating especially highly with measures thought to require analytic reasoning and, to some extent, visuospatial ability (Gustafsson 1984; Kvist and Gustafsson 2008; Lynn and Irwing 2004; Marshalek et al. 1983). The construct measured using figural matrices tests is also central to inductive reasoning, a hallmark indicator of fluid intelligence in the Cattell–Horn–Carroll model (CHC), a main structural model of intelligence (Flanagan and McDonough 2018; McGrew 2009). The tests are fully non-verbal measures intended to minimize any undesired influences from reading ability on the test scores (Cattell 1940; Raven 1941), and they are present in a multitude of test batteries, including the Wechsler tests, the Stanford–Binet test, and the Cattell Culture Fair test (Waschl and Burns 2020).

**Figure 1:**
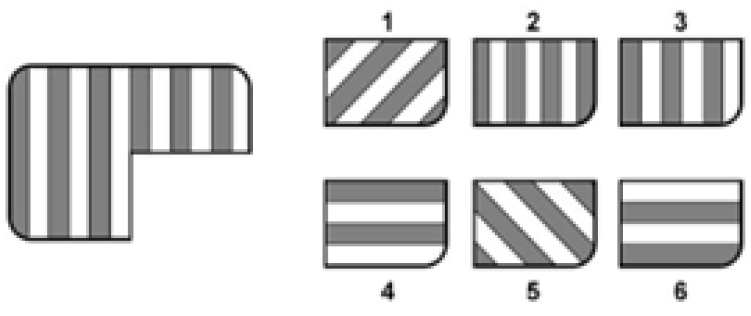
Example item from the NAF figural matrices subtest.

The NAF figural matrices test was directly modeled after Raven’s Progressive Matrices (Raven 1941), the items of which consist of presenting the test-taker with an incomplete figure and tasking them with identifying the missing piece from a number of alternatives (an example item can be found in Figure 1). At present, Raven’s is assumed to function well in Scandinavia, but there is a lack of evidence of sufficient measurement precision to make classification decisions in the Norwegian population (Helland-Riise and Martinussen 2017). This is problematic, especially since the NAF figural matrices is the fallback test whenever test-takers are unfit to take the numeracy or verbal test due to, for example, language issues or learning disabilities.

There are multiple studies that use scores from the NAFs’ GMA battery. For example, several studies have utilized it to investigate intelligence. Flynn (1987) famously used it (along with other tests) with data from 1954 to 1980 in order to support his theory on secular changes in intelligence. Sundet et al. (2004) expanded on Flynn’s study to include up to the 2002 cohort, showing stagnation in figural matrices’ mean scores and substantial distributional changes with lower standard deviations, more skew, and heavier tails, starting in the 1990s. Flynn and Shayer (2018) also used these data when studying the general stagnation and recession of measured intelligence in the western world. We have also identified several other studies that use Norwegian GMA data, and a limited review of these can be found in Appendix B.

Other studies have looked at the relationship between GMA scores and other non-psychological variables. Galloway and Pudney (2011) did a study on the relationship between GMA and the likelihood of committing a crime. Bratsberg and Rogeberg (2017) used data from the 1962–1990 birth cohorts (measured roughly in 1981–2009) to establish that the relationship between GMA and mortality is not confounded by socioeconomic status. We will make a general assessment in our discussion of how the studies used data from the GMA tests and suggest some possible issues. More studies used the GMA measures from the NAFs than the ones we have listed, but these used older data not comparable to our sample due to the Flynn effect (like, e.g., Sundet et al. 1988), using the measures in some peripheral way (e.g., Dahl et al. 2021), or they were not known to us at the time of writing.

Research done by the NAFs has found weaker correlations between their GMA measures and job performance than generally expected based on the existing literature for both conscripts (Køber et al. 2017) and candidates for military leadership training (Isaksen 2014; Norrøne 2016; Vik 2013). To better understand these results, detailed psychometric analyses at the item level are required. Recent advances in data collection via electronic testing have made this possible, and since 2011, data at the item level have become available (Skoglund et al. 2014).

### 1.2. Sex Differences in Non-Verbal Fluid Intelligence

Males have been shown to express greater general variability than females in mental ability tests (Arden and Plomin 2006; Deary et al. 2003). Several studies that have investigated the presence of sex differences in intelligence have shown mixed results. Some claim no or negligible differences (Halpern and LaMay 2000; Jensen 1998), while the meta-analysis of Lynn and Irwing (2004) found a small but consistent score advantage for males after the age of 15. Feingold (1992) found no noticeable sex differences in the mean or variance on an abstract non-verbal reasoning measure, while Strand et al. (2006, non-verbal reasoning) and Keith et al. (2011, figural matrices) found little differences in means, but they found that male performance has a greater variance. Reynolds et al. (2022), in their review, suggested that, while differences in the general level may be negligible, there might be important differences in specific abilities and subtests. They observed that, while there tend to be noticeable female advantages in processing speed, males typically perform better on visuo–spatial measures—differences in fluid reasoning, however, are inconsistent.

Reynolds et al. (2022) stated that studies of sex differences in mental ability have been overly concerned with simple distributional differences. To broaden the perspective, they suggest, amongst other things, more research on issues of measurement invariance at the item level. Some studies have already investigated measurement invariance for figural matrices or related measures with somewhat mixed results. Steinmayr et al. (2010) conducted a study of German gymnasium students, observing that the sex differences in fluid intelligence nearly vanished when the non-invariance of test scores was taken into account. Keith et al. (2008) reported that males in a US standardization sample scored higher than females on quantitative reasoning and spatial ability, which are measures regarded as close to figural matrices in the nomological network (see, e.g., Waschl and Burns 2020). Abad et al. (2004) found an advantage in solving Raven’s advanced progressive matrices for male Spanish university students, and these did not disappear when differential item functioning (DIF) was taken into account. For a sample of US children and adolescents, Lakin and Gambrell (2014) regarded levels of DIF in figural reasoning items as acceptable and so did not look at the impact of test bias on parameter estimates.

Some research has related sex differences to differences in cognitive processes involved in solving items. An excellent summary of this is the systematic review of Waschl and Burns (2020). They suggested that sex differences in inductive reasoning measurements (the CHC sub-construct encompassing figural matrices) could be attributed to male advantages in spatial ability, not in the inductive reasoning ability itself. Spatial ability is regarded as the cognitive ability with the most consistent gender differences (Hyde 2016; Reynolds et al. 2022). Lim (1994) argues that females use different problem-solving strategies that do not depend on spatial ability and that this is the cause of sex differences on figural matrices tests.

While there has long been interest in explaining sex and gender differences in test performance, it is our appraisal that most studies have concerned themselves with structural differences within the whole test battery or within substrata of that battery. Measurement invariance within individual instruments is often assumed (like, e.g., Arden and Plomin 2006; Deary et al. 2003), introducing a strong assumption of identical item performance between groups that is not necessarily reasonable. Potential patterns of item bias that seem negligible in themselves might still be a contributing factor to the existence and magnitude of observed differences if they are theoretically plausible, given existing knowledge of distributional sex differences in, for example, subtest performance (Reynolds et al. 2022) and problem-solving style (Lim 1994). This is also something that has been called for in the field (Reynolds et al. 2022). Our study contributes to the litertaure by publishing new data on sex differences in the figural matrices subtest of a well-known test battery that has been frequently used in research, though previously only on males. We also investigated whether patterns in performance differences change when potential item bias are taken into account, using modern item response modeling methods.

### 1.3. The Present Study

Our study had two main research objectives: (1) to investigate the general measurement properties of the instrument in terms of test characteristics and measurement precision and (2) to investigate item-level measurement invariance in relation to sex differences in test-level difficulty and discrimination. Both objectives sought to examine the validity of the scientific statements made using the instrument, and we appraised some of the relevant studies that have done so. To our knowledge, no previous study had examined the performance of females on the NAFs tests.

## 2. Materials and Methods

### 2.1. Sample

**Figure 2:**
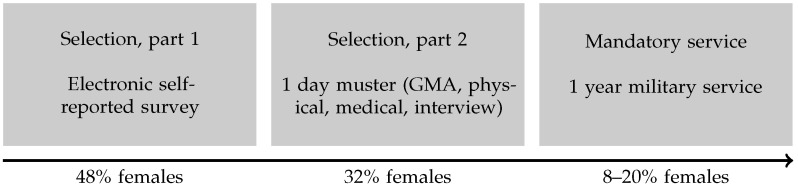
Selection process of the NAFs.

The sample consisted of 113,671 adolescents assessed as part of the muster (NO: sesjon) of the NAFs. The muster, illustrated in Figure 2, begins by requiring the entire birth cohort (N≈ 55,000–60,000 per year, with a response rate of 96–98%) to complete an electronic self-report survey (part 1). The candidates receive the survey the year they turn 17, and those found eligible based on the NAFs’ needs (n≈ 18,000–25,000 per year; see Table 1) are tested on-site approximately 2 years later (part 2). Selection is based on objective criteria like geography, sex, skills (people with certain technical certificates, etc.), and self-reported health, motivation, and physical fitness. There is some variation between years in the needs of the NAFs, but the selection for the muster is considered to be sufficiently stable. Conscription into the NAFs has always been mandatory for males, and this was also extended to females in January 2015 (Endringslov til vernepliktsloven og heimevernloven 2014).

Exceptions to the mustering protocol are postponement due to failing to achieve the standard Norwegian three-year secondary education diploma on time (having to retake subjects or exams or changing study programs) or attending 4-year technical school programs. A small number of people are tested at the age of 16 because they apply for apprenticeships specifically offered through the military (mechanics, electricians, cooks, etc.), but these were excluded from the study sample.

In the Norwegian population, females have generally higher secondary school grades, but males are more represented in technical vocational educations and have greater grade variability (NOU 2019:3 2019). According to statistics acquired from the NAFs, people that reach Stage 2 of the mustering process (thus become part of our sample) self-report higher average grades in core secondary school subjects than the general population, with smaller grade variance (an illustration can be found in Appendix A, Figure A4).

However, these sampling characteristics are not identical between sexes; deviations from the population characteristics are more pronounced for males than for females in the mean and the variance. Hence, in generalizations from secondary school grades to GMA, means and variances for the female group should be expected to be *biased downwards*. The reader is advised that any inferences about general sex differences in mental ability drawn from our study must be made with this potential bias in mind.

Data for the study were obtained by consent from the NAFs, and ethical approval was granted by the Norwegian Agency for Shared Services in Education and Research (SIKT, formerly NSD).

### 2.2. Test Administration Procedure

The session was around 60 min, and the entire battery was administered without breaks, with a subtest-wise enforced time limit, in the following order: first, the 30-item numeracy test, U4 (25 min); then, the 36-item figural matrices test, U5 (20 min); and, lastly, the 54-item word-similarities test, U6 (6 min). The situation was proctored, without interruptions, in classroom-sized rooms well suited to maintaining focus, and the participants were typically tested in groups of 20–30. The tests were given on a stationary computer with an external mouse and keyboard, typically with physical separators between computers to minimize the opportunity for cheating. Pens and paper were also provided in case test-takers wanted to take notes. The test-takers were explicitly reminded that cheating or faking on the test could lead to arrest and that they would not be able to retake the tests if they ended up wanting to join the armed forces at a later point in life.

### 2.3. Measures and Data Management

Due to its previously mentioned special importance in the conscript-selection procedure, our study concerns itself only with the figural matrices subtest of the GMA battery. The test data are item responses from the years 2011–2017 of the birth cohorts of 1992–1993 to 1998–1999 (Table 1). The items are in multiple-choice format, scored binarily, with response alternatives numbering between six and eight.

The cohort variable we used in the analysis was based on the year the participants were tested (the test year and birth year do not correspond). We trimmed each cohort to only include persons who were 19 (±1) years old at the time of testing by calculating the difference between the birth year and test year. We also excluded any observation that did not have item response data, which were about four-fifths of the 2011 cohort (the switch to computerized testing occurred during this testing cycle) but a negligible number in the other cohorts (the 2017 cohort is incomplete due to the data being acquired in the middle of that testing cycle.) A small number of observations with missing values in other variables (roughly 20 per cohort) were also excluded. Sex was defined as legal sex (in most cases, as registered at birth), and it is a binary variable acquired from the Norwegian national registry.

### 2.4. Statistical Analysis

We used descriptive statistics and item response theory to evaluate the psychometric properties of the items on the test. We utilized a unidimensional, two-parameter logistic model and evaluated the model fit in cohorts and groups, first separately by each cohort and group and then jointly across groups in each cohort. We estimated the model parameters with marginal maximum likelihood estimation using numerical quadrature (Bock and Aitkin 1981) as implemented in the R package mirt (Chalmers 2012). To evaluate model fit, we utilized the $M_{2}$ statistic, a recommended method for analyzing the fit of a model to categorical data (Tay et al. 2015), as well as the root mean square error of approximation (RMSEA) and the standardized root mean square residual (Maydeu-Olivares 2013, SRMSR). Absolute model fit with the $M_{2}$ statistic was established if the hypothesis of equality between the observed and model-implied univariate and bivariate moments for pairs of item scores was not rejected at a significance level of 0.05. Adequate model fit was established if the estimated RMSEA was lower than 0.089 and if the SRMSR was lower than 0.05, with excellent fit established at 0.05 and 0.027, respectively (for binary data, the criteria for close and excellent fit are equal, so we only used the latter term; Maydeu-Olivares and Joe 2014). We also computed IRT reliability coefficients (Kim and Feldt 2010) from configural models using the implementation of Andersson and Xin (2018).

Measurement invariance across groups was evaluated using multiple-group modeling, employing likelihood ratio tests between nested models. We evaluated configural invariance in each of the two groups in each of the cohorts, resulting in 14 models subject to evaluation. These models were evaluated for absolute and approximate fit, as described above. In cases where we established at least an approximate fit in the configural models of each cohort, we evaluated measurement invariance across groups in each of the cohorts. First, we evaluated full measurement invariance via hypothesis tests of equality between all item parameters in the groups with a significance level of .05. If this test was rejected, we conducted a partial measurement-invariance study using the two-step approach defined in (Stark et al. 2006). Their two-step procedure is summarized as follows.

Step 1:
  (a) We estimate the constrained baseline model (all item parameters set equal between groups, but the mean and variance of the latent variable were freely estimated in one group).
  (b) For each item, we removed the equality restriction for the item parameters between the groups and estimated the model. If there was a statistically significant difference to the constrained baseline model, we flagged that item as an item with a potential violation of measurement invariance.
  (c) For the items that did not show statistically significant differences, we ranked the items from the item with the highest estimated discrimination parameter to the item with the lowest estimated discrimination parameter, based on the results from the constrained baseline model. The five items with the highest estimated factor loadings were selected as anchor items.
Step 2:
  (d) We estimated the free baseline model, in which the five items from Step 1 (c) were constrained to be equal between the groups, while the remaining items were allowed to vary.
  (e) For each item, we removed the equality restriction for the item parameters between the groups and estimated the model. If there was a statistically significant difference to the constrained baseline model, we flagged that item as an item with potential violation of measurement invariance.
  (f) We obtained a model with 5+j items that were considered invariant, where *j* is the number of items identified in Step 2 (e).

With the selected models, we assessed and compared the item and scale properties in the two groups by computing item characteristic curves, test characteristic curves, item information functions, and test information functions (de Ayala 2022). The mean and variance of the latent variable in one group, in contrast to the other group, were also estimated for each cohort. To assess the impact of violations to measurement invariance, we compared the mean and variance estimates from the constrained baseline model in Step 1 (a) to the estimates of the final model in Step 2 (f) (Fischer et al. 2018).

## 3. Results

The dataset consisted of the 36 binary items of the NAF figural matrices test. Items 1–10 were excluded for having perfect or near-perfect item scores, leaving 26 items for the analysis. A table with descriptive statistics for the included items, as well as distributions of sum scores, can be found in Appendix A. The test has a disproportionate number of easy items and few hard, suggesting a possible ceiling effect. Item-total correlations will be generally higher for items of middle-high difficulty within the test. The sex variable is binary, and of the 113,671 participants, 32 % were female, with percentages varying between 28% in 2011 and 38% in 2016 (Table 1). Most cohorts had between 17,000 and 20,000 participants, with the exception of cohorts 2011 (n=3921) and 2017 (*n* = 14,011).

### 3.1. Item Response Modeling

#### 3.1.1. Configural Invariance

We analyzed configural invariance by fitting a series of unidimensional two-parameter logistic models separately by cohort, and evaluated the absolute model fit in the two groups. Our hypothesis was that the unidimensional model fit well in both groups, and from Table 2, we can see that the 95 percent confidence interval (CI) for the RMSEA was lower than the rule of thumb of 0.05 (Maydeu-Olivares and Joe 2014), indicating an excellent fitting model. SRMSR indicated that the model fit both groups at least adequately, though some of the female cohorts met the criterion for excellent fit (SRMSR ≤ 0.027). The criterion of acceptable absolute fit in each separate group was met, which justified an analysis of full and then partial measurement invariance.

IRT reliability coefficients (Kim and Feldt 2010) were computed from configural models. From Table 3, we can see that the reliability coefficients lie between .69 and .77. Coefficients are generally higher in the male groups than in the female groups (with all but three test reliability coefficients, as well as all marginal reliability coefficients, outside the 95% confidence interval of the opposite group). This is most likely because of the differences in the distribution of the latent variable and not because of differences in measurement properties of the test itself.

#### 3.1.2. Partial Invariance

The analysis of partial invariance was done in two stages, using the approach of Stark et al. (2006). In Step 1 of the analysis, we identified potential anchors among invariant items using the constrained baseline model, and we selected the five with the highest factor loadings. They were subsequently used in Step 2 as constrained referent items in the free baseline model to identify items that violated invariance.

**Figure A3:**
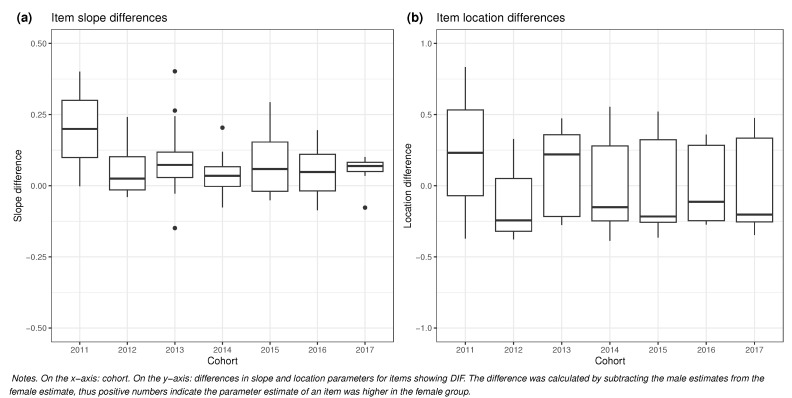
Item-parameter differences between males and females for DIF items by cohort.

From Table 4, we can see that cohorts 2013 and 2014 had by far the most non-invariant items, with 12 and 13 flagged (roughly half of the items). The 2011 cohort had the fewest at two (likely due to reduced statistical power from the smaller sample size), and the rest had between seven and nine flagged items. Generally, non-invariance tended to mostly manifest in the slope parameters (factor loadings), which were slightly steeper for females (see Figure A3 in Appendix A).

Table 5 shows the model fit measures for the different invariance models in the cohorts. Measures of absolute model fit indicated that the models fit the data well. The RMSEA indicates an excellent fit for all models in every cohort. The SRMSR indicates that all the models fit the female group better than the male (excellent fit vs. adequate fit), with the exception of the 2011 cohort.

Comparing relative fit indices based on the log-likelihood, we observed that the information criteria preferred different models. The model with partial constraints had the best fit, according to the BIC in all cohorts. The AIC preferred the configural model, with the exception of the 2014 cohort, for which it preferred the partial invariance model.

#### 3.1.3. Information Curves and Expected Score Functions

**Figure 3:**
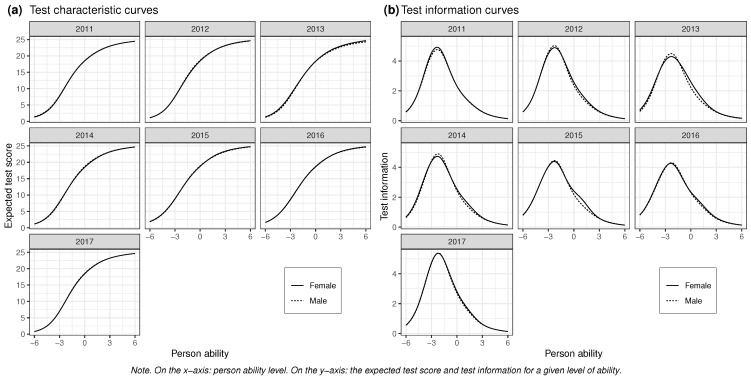
Expected test scores (**a**) and item information functions (**b**) for males and females in each cohort from the partial invariance model.

Figure 3 shows the expected score function and the test information function from the partial invariance model. The test is most informative for the lower part of the ability scale, around minus two logits, with a sharp decline moving up the scale (as the ten easiest items were removed, the decline at the lower extreme is less meaningful). This indicates that ability estimates are less certain for people with higher mental ability and that the test discriminates less well between these individuals. There is some variation between the sexes in terms of overall test information, with the peak of the curve being higher for the male group in cohorts of 2012–2014 but roughly equal in the 2015–2017 cohorts (this was also when the NAFs implemented the change in the sampling procedure), females having a higher peak only in 2011. There was some tendency suggesting that the test was more informative for higher-ability females relative to males in some of the cohorts (especially 2013 and 2015), and females on the upper part of the ability scale were also expected to have a slightly but noticeably higher test score than males in the 2013 cohort.

The standard error (SE) of the ability estimates for a given point on the ability scale was defined as $SE\left(\theta \right)=\frac{1}{\sqrt{TI(\theta )}}$, where θ is person’s ability and TI is the test information (Embretson and Reise 2000). Exact values for different parts of the ability scale can be found in Table 6. The high point on the test information curve (on the ability scale: −2.2 to −2.4 for females and −2.2 to −2.3 for males) of the NAF figural matrices test varied between 4.27 and 5.37 for females and 4.31 and 5.39 for males across the cohorts. However, this level of measurement precision only applied to people relatively low on the ability scale. At 0, the center point on the scale, the test information had been reduced to 2.32–2.81 for females and 2.18–2.69 for males, and it decreased even more as one went up the scale. These results indicate that the test does not precisely measure high-ability individuals.

Overall, the curves do not exhibit significant dissimilarity concerning the informativeness or difficulty between males and females in the test. Additionally, most of the non-invariance observed in individual items was balanced out at the test level. Consequently, a thorough examination of the item characteristic curves and item information functions provides limited additional insights into the overall measurement properties (these can be found in Appendix A).

### 3.2. Consequences of Invariance

When equality constraints on the non-invariant items are removed, there is little discernible difference in the expected score functions for males and females in any of the cohorts (Figure 3a), but differences exist for the distribution-parameter estimates. As seen in Table 7, males had noticeably higher latent means compared to females, outperforming them by between 0.24 and 0.33 logits in the constrained baseline model. We can transform this to IQ equivalents by multiplying the estimates by 15, which shows a male advantage of about 3.6–5 points on the IQ scale. Males also have noticeably higher variance estimates than the female group, in the magnitude of between 36 and 63 percent. This means that males are comparatively more represented at the extremes of the distribution.

The mean differences primarily reduced when we loosened constraints on non-invariant items in the partial invariance model (Table 7), suggesting that some of the differences could be related to invariance issues (although, for the 2013 cohort, the mean for males actually increased when non-invariance was taken into account). However, about half of the means were within the 95% confidence interval of the estimates from the other model (with 2012, 2014, and 2017 being the exceptions), so the results are ambiguous. All variance differences except for the 2013 and 2015 cohorts decreased when the constraints were loosened, although mostly within the confidence intervals of the estimates from the other model (with the constrained model for the 2014 cohort having the only interval that did not cover the estimate from the opposing model). There is a contrast between the two cohorts with the highest number of non-invariant items, with 2013’s negligible *increase* in group mean and variance differences and 2014’s (comparatively) substantial decrease in mean difference of .09 logits (1.4 IQ points) and variance difference of 0.10 (10 percentage points).

## 4. Discussion

This study utilized a large sample of Norwegian males and females to assess the properties of individual items of the commonly used NAF figural matrices test. This test and similar tests have been used in many previous studies to draw conclusions regarding trends across time and to assess differences between various groups. Our results show that the item properties of the NAF figural matrices were not always identical for males and females in our sample. Some items showed consistent differences across all the cohorts included in the study, suggesting that the differences were not due to chance. We estimated that the mean proficiency on the NAF figural matrices in our sample was slightly higher for males than females and that the variance in proficiency was also higher for males than females. This result was consistent across all cohorts, and it persisted when potential measurement bias was accounted for. Our results thus point to the existence of distributional differences in proficiency on figural matrices between males and females in the sample. At the test level, the measurement bias that we identified did not substantially change the difficulty or the measurement precision for males and females. However, accounting for measurement bias did generally reduce the mean difference estimates very slightly between males and females. We recommend fitting detailed measurement models when using this test and evaluating and accounting for potential measurement bias when drawing inferences from test scores.

### 4.1. General Measurement Properties of the Figural Matrices

#### 4.1.1. Test Reliability

Previous reporting on the measurement properties of the NAF figural matrices has mostly come from norming studies conducted by the NAFs. Thrane (1977) reported an initial test–retest reliability coefficient of .80 and a split-half coefficient of .87 for an early 24-item version of the test made in 1949. He stated that, in contrast to the other tests in the battery, the NAF figural matrices test was deliberately designed to screen at the lower part of the ability scale, and the norming sample had an observed skew of −0.5 in 1950. As we have demonstrated in our own study, this is also the general area where it discriminates the best, though when all 36 items are included, the skew in the sum scores for the 2011–2017 samples lies between −1.3 and −1.5, which is a substantial change.

Sundet et al. (1988) cited a test–retest reliability of .72 for the updated 36-item version (Notes from the psychological services of the Norwegian Armed Forces, 1956, as cited in Sundet et al. 1988). Our own observations for males (test reliability: .73–.77; marginal reliability: .72–.75; Table 3) are in line with the previous observations. In contrast, if we look at the father of the NAF figural matrices, Raven’s Standard Progressive Matrices, we find that what is most likely a lower-bound coefficient of .87 has been observed in a large sample of Swedish 12-year-olds (Gustafsson et al. 1981), but there is a lack of Norwegian reliability studies (Helland-Riise and Martinussen 2017). This suggests that the measurement precision of the U5 is below what you should expect for a Raven’s-like measure, given an appropriate population. We have also shown that reliability is substantially lower for females than for males. Considering the similar test information curves for the two groups (Figure 3b), this difference is likely due to the larger variance in ability of the male group, not differences in the measurement precision of the instrument. Still, when obtaining ability estimates of individuals or groups with such tests, it would be prudent to adjust for reliability in the groups separately.

#### 4.1.2. Test Validity and Scaling

The issues we have identified with the figural matrices test impact the predictive value of the overall composite GMA scores, and we believe this is likely to be a contributing factor to the lower-than-expected predictive validity observed in other studies (Isaksen 2014; Køber et al. 2017; Norrøne 2016; Vik 2013). Although the GMA composite score would most likely meet the normality assumption of a multiple-regression model, undesirable scale properties like a ceiling effect in a measure could still suppress correlations, and this is not solved using standardization procedures (McDonald 1999). While our study did not seek to answer how or whether the undesirable properties of the figural matrices disappear in the composite GMA variable, it is worth keeping the added uncertainty in mind when using the GMA scores in research and selection. Using the NAF figural matrices test on its own for classification outside the lower parts of the scale should be avoided.

A number of studies cited Sundet et al. (1988) or Sundet et al. (2004) as the basis for their scaling or used similar procedures. These approaches to creating the GMA composite scores are to standardize and center the raw scores on each subtest, combine them (unweighted), and transform them into stanine scores, which are used in the analysis. In the paper of Sundet et al. (2004), they often transformed scores into IQ equivalents, with a mean of 100 and a standard deviation of 15, based on norms from 1954. This kind of linear transformation seems to have eventually cemented itself as the ”conventional” method for treating the measures (Bratsberg and Rogeberg 2018). (Studies using variants include Bjerkedal et al. 2007; Black et al. 2007, 2009, 2011; Bratsberg and Rogeberg 2017; Kristensen and Bjerkedal 2007; Sundet et al. 2008; Sundet et al. 2005; Sundet et al. 2010—for a short review of these, see Appendix B.) Many studies using data spanning a large number of years, like those investigating generational changes or the Flynn effect, are likely to see limited comparability because of the measurement issues inherent in the instrument that we have identified. For example, inferences about relations between the scores of fathers and sons might be problematic if the sons come from a generation with a more pronounced ceiling effect, as the decrease in variance will suppress correlations. Researchers need to account for this in their interpretations. There might be some merit to approaches like, for example, that of Bratsberg and Rogeberg (2017), who partitioned the sample by the year the Flynn effect ended (birth cohort 1975), essentially treating them as separate populations.

We would also be careful using strict cut-offs on the NAF GMA scale itself, and especially the figural matrices subtest. We identified two studies, Galloway and Pudney (2011) and Flynn and Shayer (2018), that used strict cut-offs to split the scale into high- and low-scoring persons. Galloway and Pudney (2011) stated a cut-off at the sixth stanine of the GMA composite, with a main interest in the lower part of the scale. The lower threshold for the sixth stanine was supposed to be half a standard deviation above the mean—in our study samples, for the NAF figural matrices, this roughly equaled a sum score of 30, but if they were scoring according to the norms of 1954 (the ”conventional” method), it would be lower. Flynn and Shayer (2018) used a sum score of 30 on the NAF figural matrices as a cut-off (which is also half a standard deviation above the sample means cf. Sundet et al. 2004), with a main interest in the upper part of the scale. In our study, for the same level of ability (in logits: males, 0.6–0.9; females, 0.6–0.8^1^), we observed standard errors of measurement of 0.71–0.82 and 0.69–0.78, respectively—error intervals essentially nearing the sample means. If generalizable to samples from the 1990s, this is not great precision, and, as per earlier remarks, the general tenability of categorizing persons around these levels on the scale should be considered highly uncertain. Hence, results from the use of this approach must be interpreted with caution.

Studies that fail to take error of measurement into account risk a distortion of measurement at the extremes of the scale (McDonald 1999), and in the case of the NAF figural matrices, this is at the top. It is difficult to ascertain the exact magnitude of impact of this on the studies that use composite scores from the NAF GMA battery, especially when the object of interest is in the entire scale, other than to suggest a possible underestimation of any correlations between the composite scores and other variables (validity- and reliability-related estimates from similar test batteries might also not be exactly mirrored in NAF GMA data). What we can say is that researchers wanting to make inferences about performance on the upper part of the GMA scale should bear in mind the uncertainties, and for performance on the NAF figural matrices subtest itself, there will be a weak empirical basis on which to make any conclusions. This would not necessarily be obvious from only considering classical test-theory statistics (McDonald 1999).

### 4.2. Sex Differences on Non-Verbal Fluid Intelligence Measures

Our other purpose of this study was to investigate how the NAF figural matrices measure males and females. Removing restrictions on the parameters of items that did not demonstrate invariance improved the model fit. Notably, all the models had a closer fit in the female group than the male group in almost all cohorts, and all models fit better in the female group than the male group. The better fit of most of the models in the female groups (Table 5) could be because the larger male group was somewhat more diverse in terms of social background and vocation.

When it comes to differences in distribution, our findings suggest that there is noticeably higher male variance in performance on figural matrices in our sample, by between 36 and 63 percent when assuming measurement invariance and 32 and 69 percent when adjusting for violations of invariance. As the reduction in variance between models tended to be negligible, the greater male variability we observed cannot be attributed to invariance issues within the NAF figural matrices. Differences in average performance were also substantial, with males outperforming females by between 3.6 and 5 IQ points, and 2.2 and 4.8 IQ points in the respective models (Table 7). Like Steinmayr et al. (2010), we also observed a tendency for sex differences in means to become smaller when non-invariance was taken into account. They suggested that the observed sex differences in figural reasoning could stem from intelligence being underestimated in the male group at time of selection due to boys maturing later than girls. We would argue that we do not have this particular weakness in our study, as unlike with university students, our sampling was less based on criteria like grades. Other authors have suggested that inconsistencies in observed gender effects on fluid intelligence measurement have to do with variation in instrument characteristics between studies (Colom and García-López 2002). In our study, we experienced these kinds of inconsistencies between years. The go-to explanation for us would be the nature of the sample changing, possibly because of changes in general motivation to join the armed forces, as well as the changing recruitment procedure of the NAFs. However, we observed a similar pattern of sex differences both before and after a major policy change. Explaining the anomaly of the 2013 cohort (in which the male advantage increased marginally after adjusting for DIF) is not straightforward, but it could be related to the previously mentioned varying sampling criteria. Inconsistent findings have previously been suggested to be related to fundamental differences between sample makeups (Waschl and Burns 2020). However, male–female differences in average grades for mustered and conscripted individuals have been observed to be relatively consistent between the 2013–2016 cohorts (Køber 2016). Feng et al. (2007) found that differences in spatial ability were reduced with exercise in visual monitoring and attention. Comparable environmental changes could very well be present between years in Norwegian society, and if this is the case, there is little reason to assume that it would not be reflected in our relatively large samples.

We have not considered the relationship between item content and the non-invariance observed in some items. However, this does not mean that individual item characteristics are without interest. Some research has found that males could be expected to do disproportionally better with more geometrically complex figural matrix items suggested to be medium to high in difficulty (Arendasy and Sommer 2012), and some studies of English secondary school students (Mackintosh and Bennett 2005; Plaisted et al. 2011) have found that males had an easier time with items containing addition/subtraction rules and distribution of two values rules (also medium-high in expected difficulty, following the taxonomy of Carpenter et al. 1990). In comparison, the items in our study that we identified as having possible measurement bias in five or more cohorts (Table 4) were items 19, 21, 23, 28, 29, 31, 32, and 34, which would mostly be in the mid-range spectrum of a comparable scale. Even though the test lacks items measuring the very top of the ability spectrum, the male advantage persists. One explanation of higher male motivation lessening the impact of test fatigue could be postulated from the very substantial differences in motivation in the birth cohorts. However, the differences in motivation might only be minor—data have only been published for the 2016 and 2017 cohorts, but here, 4% of males and 5% of females who reached the testing phase (selection part two; Figure 2) self-reported a low motivation for military service (Køber 2020). There is a chance that differences in problem-solving strategies (Lim 1994) could, for example, lead to females spending too much time and effort on the easy items, thus underperforming on (or not reaching) the latter part of the test.

### 4.3. Limitations of the Study

Like for previous studies, the problems with measurement that we have pointed out in this article also affect our own study. The exact impact is hard to quantify, but one would think that it could have a larger effect on the group assumed to have heavier tails on latent ability, and test information curves (Figure 3b) indeed show that the NAF figural matrices test is marginally less able to discriminate between males than females who are above average on the scale. There could also be more uncertainty around the parameter estimates of that group.

While our findings might provide some generalizable insights into how males and females perform on this core measure of mental ability, there are several factors that should be taken into account when interpreting our results.

**Figure A4:**
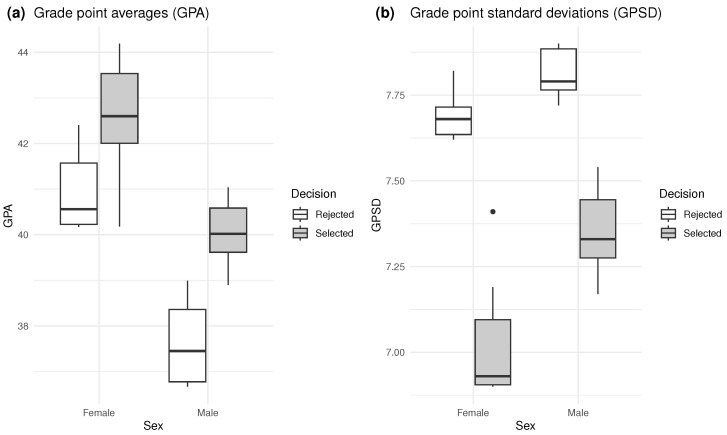

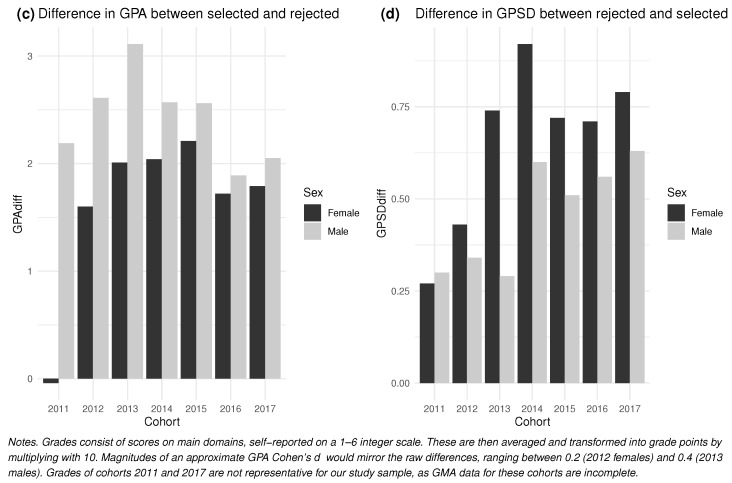
Grade patterns in the sample and population.

Firstly, while the sample sizes were considerable, this was not a proper population study. The sex distribution was quite skewed, with males over-represented in every cohort (Table 1). Sex differences in self-reported grades in the sample followed the general patterns of the population, but *magnitudes of sample-population differences* deviate somewhat between sexes (Figure A4). This should be kept in mind when interpreting the observed differences in performance on the figural matrices test.

Secondly, there is the assumption that some sampling bias exists with regards to who exactly reaches the assessment phase due to the self-selection effect brought up earlier in the article. While the difference in the motivation for reaching the selection phase is not necessarily mirrored in the test samples (as reported by Køber 2020), we cannot strictly rule out a substantial difference in test motivation in the cohorts for which data have not been published. However, it does support the internal validity of the study; thus, the sex differences we observed in measurement characteristics and distributions of estimated ability are credible within the sample.

Thirdly, the NAFs have a substantially lower demand for personnel than there are available adolescents; thus, a large number of candidates are a priori excluded from consideration, and the needs also vary between years along with organizational changes and the rapid development of military technology and changing threats (although eventual changes tend to be minor). This might be a threat to the comparability between cohorts.

Lastly, while invariance was mainly established for items using statistical procedures, not theory, there is some inherent uncertainty as to whether items classified as invariant in the procedure truly measure equivalently. As shown by Lopez Rivas et al. (2009), including non-invariant items as referents could drastically reduce the power to detect small DIF (which should be reasonable to expect for figural matrices), and while the two-stage approach of Stark et al. (2006) can reduce the risk by identifying referents using the overly sensitive constrained baseline model, misidentification cannot truly be ruled out so long as a causal explanation for non-invariance has not been established.

When all of this is said, we believe that, even though the samples deviate to a degree from the population, these deviations seem to be present in both groups. While generalizing raw sex differences from sample to population is problematic (as mentioned in Section 2.1), there is little reason to assume that *violations of measurement invariance* and its impact on ability estimates that exist in the population will not be reflected in our relatively large sample.

## 5. Conclusions

On the test level, we have found that NAF figural matrices function similarly for both males and females. Measurement precision is low at the upper part of the ability scale, but this applies to both males and females. In our sample, there are sex differences in ability, with males scoring higher on average and having a higher variance. This should be expected, given the sample characteristics, in which females’ average proficiency and variability are likely to both be biased downwards. The differences in distributional characteristics did not go away after adjusting for measurement non-invariance. Because of the poor overall measurement precision of the test, making decisions about individuals based on the figural matrices alone is not advisable.

A strength of this study is that we used data that are arguably more diverse than your average psychology study sample. A way to check the robustness of our results would be to conduct a study with a larger focus on group comparability either by acquiring reasonable background variables to use as covariates in a model or by trimming the sample, removing participants that do not have matching patterns of background variables in the other group. Future studies should investigate the concrete impact that the measurement issues we have identified have on the GMA composite scores used in previous intelligence research. With regards to sex differences, we chose not to deal with the issue of spatial ability, but since there are few large-scale studies on this in the Norwegian population, it could be a possible future direction to take. Making the connection between group differences and perceptual complexity is also a viable way going forward. This can, for instance, be done by applying models with item or item-group predictors like the linear logistic test model (LLTM, using the approach of, e.g., Janssen et al. 2004), classifying items according to the Carpenter et al. (1990) taxonomy of item complexity for figural matrices, with some perceptual facets like, e.g., those described by Primi (2001) or Arendasy and Sommer (2005).

A wider implication of our findings is that, if measurement precision is a problem in the *Norwegian* data because instruments have not been updated along with the Flynn effect, it might also be a problem in data from comparable countries, and this should be investigated by relevant researchers. While updating the instruments will naturally have consequences for comparability between years, the trade-off with *measurement quality* might not be worth the easier interpretability. For the sake of science, we would urge test owners who intend to update their instruments to include referent items from older versions in order to allow for test equating so that at least some comparability can be preserved.

The concrete takeaway from this study is that previous studies using the Norwegian GMA data must be interpreted with more caution than has been the case so far in the field of intelligence. Our view is also that figural matrices tests should measure males and females equally fairly and that an instrument in poor condition is poor for both sexes.

## Figures and Tables

**Table 1:** Sex distribution of the sample cohorts.

Cohort	2011	2012	2013	2014	2015	2016	2017
*n*	3921	18,238	19,597	17,668	18,649	17,569	14,011
Female	28%	27%	31%	31%	34%	38%	34%
Male	72%	73%	69%	69%	66%	62%	66%
Birth year	1992–1993	1993–1994	1994–1995	1995–1996	1996–1997	1997–1998	1998–1999

**Table 2:** $M_{2}$ tests of the configural model for each sex in all cohorts.

Cohort	Group	$M_{2}$	df	*p*	RMSEA (2.5%, 97.5%)	SRMSR
2011	Female	460.999	299	.001>	0.022 (0.017, 0.027)	0.039
	Male	719.818	299	.001>	0.022 (0.020, 0.025)	0.035
2012	Female	878.534	299	.001>	0.020 (0.018, 0.022)	0.028
	Male	2302.478	299	.001>	0.022 (0.021, 0.024)	0.034
2013	Female	961.704	299	.001>	0.019 (0.017, 0.021)	0.025
	Male	2433.951	299	.001>	0.023 (0.022, 0.024)	0.031
2014	Female	982.257	299	.001>	0.020 (0.018, 0.022)	0.026
	Male	2188.407	299	.001>	0.022 (0.021, 0.023)	0.030
2015	Female	836.257	299	.001>	0.016 (0.015, 0.018)	0.022
	Male	1969.806	299	.001>	0.021 (0.020, 0.022)	0.028
2016	Female	869.110	299	.001>	0.017 (0.015, 0.018)	0.022
	Male	1931.236	299	.001>	0.022 (0.021, 0.023)	0.030
2017	Female	818.726	299	.001>	0.019 (0.017, 0.021)	0.026
	Male	1656.481	299	.001>	0.021 (0.020, 0.022)	0.031

Notes. RMSEA = root mean square error of approximation, SRMSR = standardized root mean square residual. 2.5% and 97.5% is the 95% confidence interval of the RMSEA.

**Table 3:** IRT reliability coefficients with confidence intervals.

Cohort	Test Reliability		Marginal Reliability	
	Female	Male	Female	Male
2011	.70 (.58, .83)	.77 (.74, .79)	.70 (.68, .73)	.75 (.73, .76)
2012	.72 (.70, .73)	.77 (.72, .81)	.72 (.71, .73)	.75 (.74, .75)
2013	.70 (.69, .72)	.77 (.69, .85)	.71 (.70, .72)	.75 (.74, .75)
2014	.71 (.69, .73)	.76 (.75, .77)	.71 (.70, .72)	.74 (.73, .75)
2015	.69 (.68, .71)	.73 (.72, .75)	.70 (.69, .71)	.72 (.71, .73)
2016	.69 (.67, .71)	.74 (.73, .76)	.70 (.69, .71)	.73 (.72, .74)
2017	.73 (.71, .76)	.77 (.72, .81)	.73 (.72, .74)	.75 (.74, .75)

Notes. IRT = item response theory. From left to right: IRT test reliability, IRT marginal reliability. The 95% confidence interval (CI) of the method is in parentheses. The test reliability estimates for 2011 females, and 2013 and 2017 males, had CIs overlapping the point estimate of the other group.

**Table 4:** Non-invariant items in each cohort.

Item	2011	2012	2013	2014	2015	2016	2017	DIF
11			o	o				2
12								0
13								0
14								0
15			o					1
16								0
17			o	o			o	3
18								0
19			o	o	o	o	o	5
20								0
21		o		o	o		o	4
22								0
23	o	o	o	o	o	o	o	7
24								0
25		o	o	o			o	4
26			o					1
27								0
28			o					1
29	o	o	o	o	o	o	o	7
30				o		o		2
31		o	o	o	o	o	o	6
32		o	o	o	o	o	o	6
33				o	o			2
34		o	o	o	o	o	o	6
35								0
36			o					1
DIF	2	7	13	12	8	7	9	

Notes. DIF = differential item functioning. Circles indicate non-invariant items in the free baseline stage (Step 2) at a Bonferroni-corrected .05 significance level.

**Table 5:** Fit statistics for the constrained model, the model with partial constraints, and the configural model.

Cohort	Model	AIC	BIC	RMSEA (2.5%, 97.5%)	SRMSR.F	SRMSR.M
2011	Constrained	73,262	73,601	0.016 (0.015, 0.018)	0.042	0.036
	Partial	73,222	**73,586**	0.016 (0.014, 0.017)	0.041	0.036
	Configural	**73,220**	73,872	0.016 (0.014, 0.017)	0.039	0.035
2012	Constrained	339,083	339,505	0.016 (0.015, 0.017)	0.029	0.035
	Partial	338,807	**339,338**	0.015 (0.015, 0.016)	0.029	0.034
	Configural	**338,724**	339,536	0.015 (0.015, 0.016)	0.028	0.034
2013	Constrained	375,974	376,401	0.016 (0.016, 0.017)	0.026	0.033
	Partial	375,465	**376,098**	0.015 (0.015, 0.016)	0.026	0.032
	Configural	**375,426**	376,249	0.015 (0.015, 0.016)	0.025	0.031
2014	Constrained	341,680	342,102	0.016 (0.015, 0.017)	0.027	0.031
	Partial	**341,319**	**341,929**	0.015 (0.014, 0.016)	0.026	0.031
	Configural	341,334	342,147	0.015 (0.015, 0.016)	0.026	0.030
2015	Constrained	351,901	352,326	0.015 (0.014, 0.015)	0.023	0.029
	Partial	351,566	**352,118**	0.014 (0.013, 0.014)	0.022	0.028
	Configural	**351,540**	352,360	0.014 (0.013, 0.014)	0.022	0.028
2016	Constrained	331,109	331,530	0.015 (0.014, 0.015)	0.023	0.031
	Partial	330,872	**331,403**	0.014 (0.014, 0.015)	0.022	0.031
	Configural	**33,0845**	331,657	0.014 (0.014, 0.015)	0.022	0.030
2017	Constrained	278,244	278,655	0.015 (0.015, 0.016)	0.026	0.033
	Partial	277,944	**278,492**	0.014 (0.014, 0.015)	0.025	0.032
	Configural	**277,938**	278,730	0.014 (0.014, 0.015)	0.026	0.031

Notes. AIC = Akaike information criterion; BIC = Bayesian information criterion. F = female; M = male. 2.5% and 97.5% represents the 95% confidence interval of the RMSEA. Boldface indicates the model with the lowest AIC or BIC in each cohort.

**Table 6:** Test information and standard errors for different levels of latent person ability θ in each group.

	−6	−5	−4	−3	−2	−1	0	1	2	3
TI.F	0.56–0.82	1.33–1.66	2.89–3.05	4.00–4.74	4.18–5.32	3.28–4.19	2.32–2.81	1.53–1.86	0.99–1.14	0.59–0.64
TI.M	0.55–0.81	1.31–1.62	2.81–2.99	3.97–4.71	4.25–5.34	3.32–4.11	2.18–2.69	1.44–1.75	0.93–1.07	0.58–0.61
SE.F	1.11–1.34	0.78–0.87	0.57–0.59	0.46–0.50	0.43–0.49	0.49–0.55	0.60–0.66	0.73–0.81	0.94–1.01	1.25–1.30
SE.M	1.11–1.35	0.79–0.87	0.58–0.60	0.46–0.50	0.43–0.49	0.49–0.55	0.61–0.68	0.76–0.83	0.97–1.04	1.28–1.32

Notes. TI = test information; SE = standard error; M = male; F = female. Ranges are between cohorts.

**Table 7:** Means and variances of males relative to females from the constrained baseline and the partial invariance model.

Cohort	Means		Variances		DIF
	Constr. (2.5%, 97.5%)	Partial (2.5%, 97.5%)	Constr. (2.5%, 97.5%)	Partial (2.5%, 97.5%)	
2011	0.32 (0.22, 0.41)	0.30 (0.20, 0.39)	1.62 (1.39, 1.85)	1.59 (1.36, 1.82)	2
2012	0.33 (0.28, 0.37)	0.25 (0.20, 0.30)	1.52 (1.42, 1.62)	1.51 (1.40, 1.63)	7
2013	0.30 (0.25, 0.34)	0.32 (0.27, 0.37)	1.63 (1.53, 1.73)	1.69 (1.55, 1.82)	13
2014	0.24 (0.20, 0.29)	0.15 (0.10, 0.20)	1.45 (1.36, 1.55)	1.35 (1.23, 1.46)	12
2015	0.27 (0.23, 0.31)	0.24 (0.19, 0.28)	1.36 (1.28, 1.44)	1.38 (1.27, 1.48)	8
2016	0.32 (0.28, 0.36)	0.28 (0.24, 0.33)	1.48 (1.39, 1.57)	1.42 (1.32, 1.52)	7
2017	0.25 (0.21, 0.29)	0.19 (0.14, 0.24)	1.36 (1.27, 1.45)	1.32 (1.21, 1.42)	9

Notes. Constr. = constrained baseline model; partial = partial invariance model. The means and variances for the female group were fixed to 0 and 1 in the estimation, and the difference between the groups is thus equal to the mean in the male group. The numbers in parentheses indicate the 95% confidence interval of the method. The DIF column counts the number of DIF items from the partial invariance modeling (Step 2) in that cohort.

**Figure A1:**
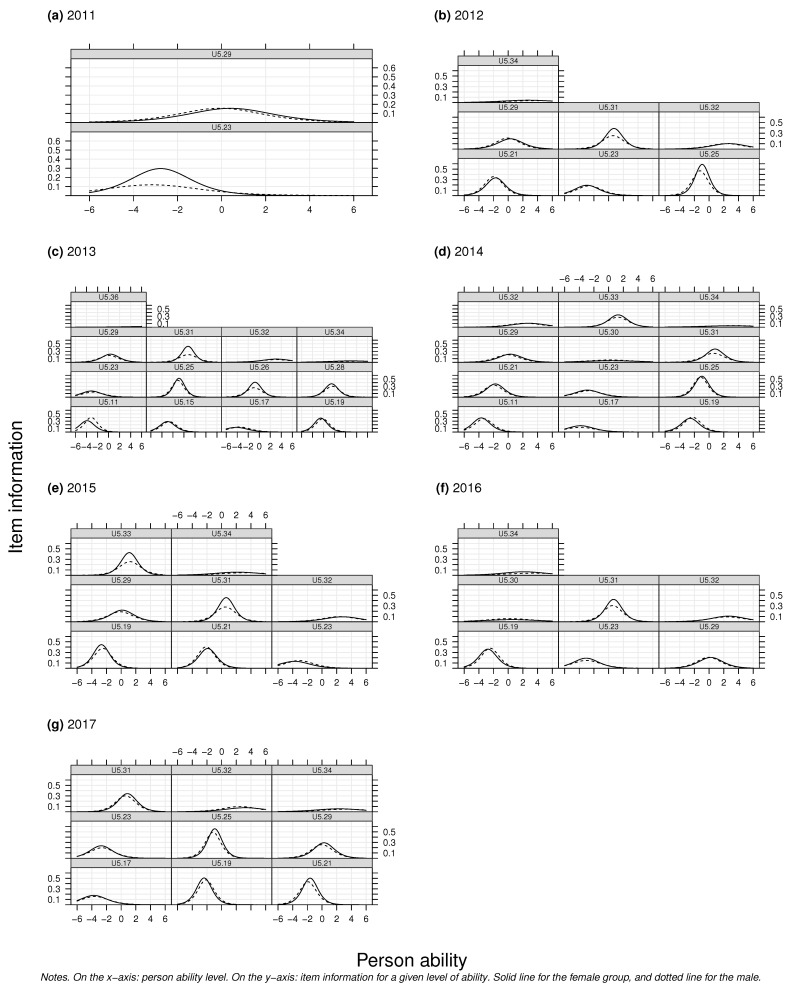
Item information curves for the non-invariant items for men and women in each cohort.

**Figure A2:**
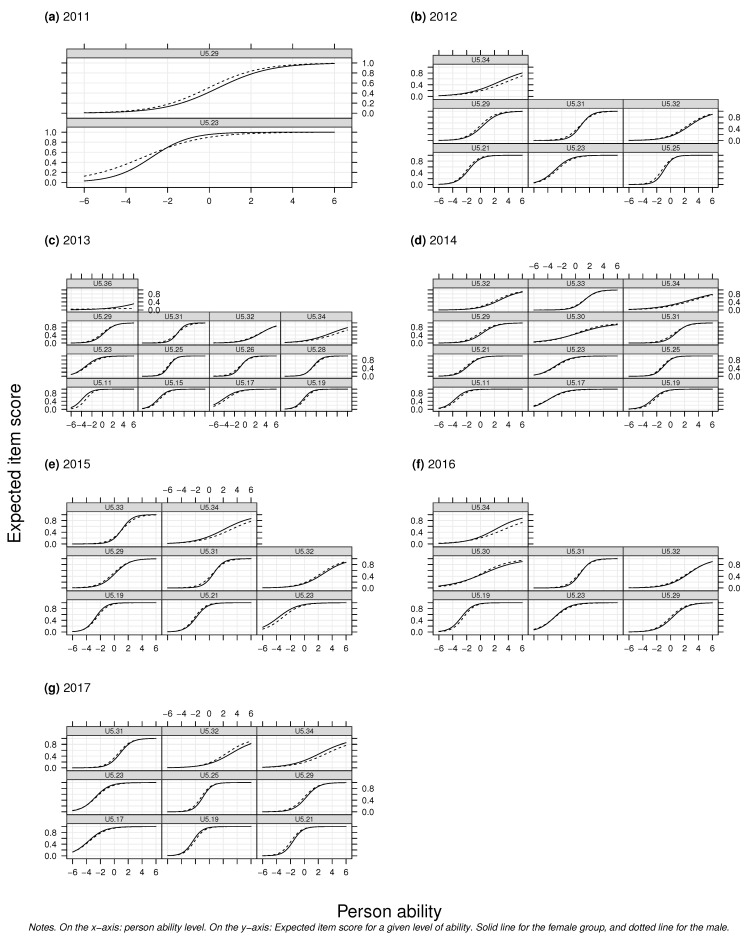
Item characteristic curves for the non-invariant items for men and women in each cohort.

**Figure A5:**
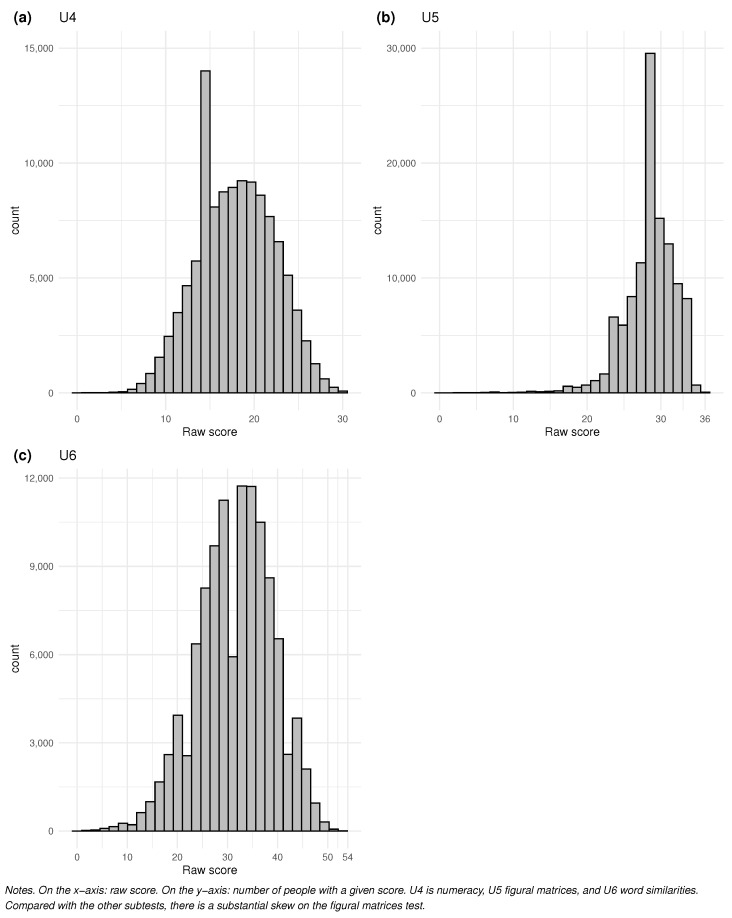
Distribution of raw scores for each subtest in all cohorts combined.

## Data Availability

The datasets presented in this article are not readily available because of restrictions from the data owner.

## Note

1 If we multiply by 15 and add 100 to get IQ equivalents, males have estimates of 109–113.5 with standard errors of 10.65–12.30 IQ points; and females have estimates of 109–112 with standard errors of 10.35–11.70 IQ points.

## Abbreviations

The following abbreviations are used in this manuscript:

GMA	General mental ability
NAFs	Norwegian Armed Forces
IRT	Item response theory
DIF	Differential item functioning
CHC	Cattell–Horn–Carroll
SIKT	Norwegian Agency for Shared Services in Education and Research
AIC	Akaike information criterion
BIC	Bayesian information criterion
CI	Confidence interval
GPA	Grade point averages
GPSD	Grade point standard deviations

## Appendix A

### Appendix A.1. Invariance Tests

**Table A1:** Non-invariant items across cohorts from Stage 1: the constrained baseline model (equality constraints on all parameters).

Item	2011	2012	2013	2014	2015	2016	2017	DIF
11			o	o			o	3
12								0
13								0
14								0
15		o	o					2
16								0
17		o	o	o			o	4
18								0
19		o	o	o	o	o	o	6
20								0
21		o		o	o	o	o	5
22		o						1
23	o	o	o	o	o	o	o	7
24								0
25		o	o	o			o	4
26			o					1
27			o					1
28		o	o	o		o	o	5
29	o	o	o	o	o	o	o	7
30						o		1
31		o	o	o	o	o	o	6
32		o	o	o	o	o	o	6
33					o	o		2
34		o	o	o	o	o	o	6
35								0
36			o					1
DIF	2	12	14	11	8	10	11	

Note. Circles indicate non-invariant items in the constrained baseline stage (Step 1) at a Bonferroni-corrected .05 significance level.

In Table A1, we can see that two items were flagged as non-invariant in the 2011 cohort (the smallest sample). For the rest of the cohorts, between 8 and 14 were identified. Items 23 and 29 were flagged in all cohorts, while items 19, 31, 32, and 34 were flagged in the six full-size cohorts. Items 21 and 28 were flagged in five cohorts, and items 17 and 25 were flagged in four.

The anchors can be found in Table A2. These were mostly located around the start-point and mid-point of the test (no items after item 27 were chosen as anchors). From the table, we can see that cohorts 2013 and 2014 had by far the most non-invariant items, with 12 and 13 flagged (roughly half of the items), while the 2011 cohort had the fewest at 2 (likely due to reduced statistical power from the smaller sample size), and the rest had between 7 and 9 flagged items. Items 11, 21, 25, and 26 were flagged as anchors in some cohorts but as non-invariant in others—essentially, items with high factor loadings could also fail invariance tests, emphasizing that invariance could be small in magnitude.

**Table A2:** Non-invariant items and anchors across cohorts.

Item	2011	2012	2013	2014	2015	2016	2017	DIF	Anchors
11	A	A	o	o	A			2	3
12	A	A	A	A	A	A	A	0	7
13		A						0	1
14								0	0
15			o					1	0
16								0	0
17			o	o			o	3	0
18			A	A	A	A	A	0	5
19			o	o	o	o	o	5	0
20	A		A	A			A	0	4
21		o	A	o	o		o	4	1
22								0	0
23	o	o	o	o	o	o	o	7	0
24		A	A	A				0	3
25	A	o	o	o	A	A	o	4	3
26			o			A	A	1	2
27	A	A		A	A	A	A	0	6
28			o					1	0
29	o	o	o	o	o	o	o	7	0
30				o		o		2	0
31		o	o	o	o	o	o	6	0
32		o	o	o	o	o	o	6	0
33				o	o			2	0
34		o	o	o	o	o	o	6	0
35								0	0
36			o					1	0
DIF	2	7	13	12	8	7	9		

Notes. “A" indicates that the item was chosen as an anchor in Step 1. Circles indicate non-invariant items in the free baseline stage (Step 2) at a Bonferroni-corrected .05 significance level.

Figure A3 shows differences in slope (or factor loadings (**a**) and location (or thresholds (**b**) for DIF items between males and females. The slopes of the DIF items tend to be slightly higher for females, but differences in location parameters have more ambiguous patterns.

### Appendix A.2. Descriptive Statistics, Score Distributions, and Sample Characteristics

Statistics on self-reported secondary school grades in the the sample and population were kindly provided by the NAFs, and they are presented in Figure A4. Looking at the distribution of grade point averages (GPAs) reveals that the generally higher female secondary school grades in the population are also reflected in the sample (Figure A4a,b). However, the positive *difference* in GPAs is greater for males than for females (Figure A4c); thus, it is reasonable to assume that abler males are disproportionally selected. Variation (in the form of grade point standard deviations; GPSDs) is also lower in the sample than in the population for both groups, but here, the difference is greater in the female group (Figure A4d). Hence, estimated GMA means and variances for the female group should also be expected to be biased downwards.

Table A3 shows descriptives and item-total correlations for each cohort, Table A4 and Table A5 describe the score distribution of each cohort for males and females, and Figure A5 shows the distributions of raw scores for all three subtests of the NAF GMA battery. From looking at the statistics, we see that most participants managed to answer the early items correctly, with a proportion correct in the 90% area. There is a gradual decrease as we progress later into the test, and from item 30, the proportions dip below .50. We can see from the item descriptives alone that the test has a disproportionate number of easy items and few hard, suggesting a possible ceiling effect (this can also be seen in the shape of the raw score distribution; Figure A5b). Item-total correlations vary between a low .09 for item 36 and a medium .54 for item 25, with the highest correlations generally in items with a lower proportion correct (of middle-high difficulty within the test). Responses on the very last item were practically uncorrelated with the total score, suggesting that this item is particularly poor or was rarely reached. Due to the sample-population issues mentioned in Section 2.1 in reference to Figure A4, making bombastic statements about between-cohort differences (Table A4 and Table A5) without theory for support is questionable, which is why we have avoided doing so.

**Table A3:** Descriptive statistics and item-total correlations.

Item	M	SD	Item–Total
11	.97–.97	.16–.18	.27–.34
12	.98–.98	.13–.15	.24–.32
13	.96–.97	.17–.19	.23–.32
14	.93–.95	.22–.26	.31–.35
15	.92–.94	.24–.27	.32–.38
16	.93–.94	.24–.26	.22–.29
17	.93–.94	.23–.25	.22–.32
18	.91–.93	.25–.28	.36–.41
19	.90–.94	.25–.29	.37–.45
20	.94–.95	.22–.24	.30–.38
21	.86–.89	.31–.35	.43–.46
22	.88–.90	.30–.33	.24–.29
23	.89–.91	.28–.31	.31–.36
24	.91–.93	.26–.29	.35–.40
25	.77–.81	.39–.42	.51–.54
26	.71–.75	.44–.45	.49–.51
27	.91–.93	.25–.29	.42–.49
28	.63–.67	.47–.48	.50–.53
29	.51–.53	.50–.50	.45–.49
30	.47–.52	.50–.50	.35–.37
31	.38–.41	.49–.49	.47–.50
32	.19–.21	.40–.41	.30–.33
33	.26–.30	.44–.46	.43–.46
34	.22–.24	.42–.43	.25–.29
35	.23–.25	.42–.43	.31–.34
36	.06–.07	.24–.25	.09–.13

Notes. M and SD indicate proportion correct and standard deviations. The former is bounded between 0 and 1 and the latter between 0 (no variation) and .7 (half correct and half incorrect). Ranges are between cohorts.

**Table A4:** Descriptive statistics for the raw score distribution of the figural matrices test—males.

Cohort	M	SD	Skewness	Kurtosis	Q05	Q95	Min	Max	*n*
2011	28.55	3.60	−1.61	8.88	23	33	5	36	2820
2012	28.57	3.63	−1.66	9.04	22	33	0	36	13,251
2013	28.35	3.66	−1.58	8.51	22	33	0	36	13,953
2014	28.45	3.55	−1.54	8.77	22	33	1	36	12,621
2015	28.89	3.25	−1.44	8.80	23	33	0	36	12,854
2016	28.83	3.35	−1.48	8.95	23	33	1	36	11,437
2017	28.42	3.61	−1.62	9.13	22	33	1	36	10,065
All	28.58	3.53	−1.59	9.04	23	33	0	36	77,335
Range	0.54	0.41	0.22	0.62					

Note. The table also includes observations that were excluded from the main analysis (as per Section 2.3).

**Table A5:** Descriptive statistics for the raw score distribution of the figural matrices test—females.

Cohort	M	SD	Skewness	Kurtosis	Q05	Q95	Min	Max	*n*
2011	28.10	3.10	−1.13	7.14	23	32	5	35	1105
2012	28.03	3.23	−1.22	7.66	23	32	2	36	4992
2013	27.94	3.11	−1.05	6.86	23	32	3	36	6101
2014	28.08	3.11	−1.09	7.16	23	33	3	36	5677
2015	28.44	3.01	−1.01	6.48	23	33	5	36	6710
2016	28.27	3.01	−0.97	6.66	23	33	0	35	6757
2017	27.97	3.29	−1.19	7.28	22	33	3	35	4886
All	28.14	3.12	−1.09	7.05	23	33	0	36	36,336
Range	0.50	0.28	0.25	1.18					

Note. The table also includes observations that were excluded from the main analysis (as per Section 2.3).

## Appendix B. Other Studies Using the GMA Measures of the Norwegian Armed Forces

Some studies use GMA scores to make statements about the relation of intelligence to family size and birth order. Using the data of Sundet et al. (2004), studies have looked at differences in GMA scores by family size (Black et al. 2007; Sundet et al. 2008) and whether age differences exist within sibling groups (Sundet et al. 2010). Data from test years 1984–2004 were used to make statements on the relationship between intelligence and siblingship (Bjerkedal et al. 2007; Black et al. 2011). Black et al. (2009) also did a study on the intergenerational transmission of GMA scores, using data of fathers (measured in 1952–1953) and sons (measured in 1984–2005). Sundet et al. (2005) conducted a study of a few thousand twins, using parts of the data of Sundet et al. (2004), between 1967 and 1979 (measured in roughly 1986–1998). In a notable study, Bratsberg and Rogeberg (2018) used data on birth cohorts of 1962–1991 (measured in roughly 1981–2010) to make statements on the heritability of intelligence.
